# General practitioner provision of preventive child health care: analysis of routine consultation data

**DOI:** 10.1186/1471-2296-13-73

**Published:** 2012-08-03

**Authors:** Rachael Wood, Philip Wilson

**Affiliations:** 1 Centre for Population Health Sciences, University of Edinburgh, Teviot Place, Edinburgh, EH8 9AG, UK; 2 Public Health Medicine, Information Services Division, NHS National Services Scotland, Gyle Square, 1 South Gyle Crescent, Edinburgh, EH12 9EB, UK; 3 General Practice and Primary Care, Institute of Health and Wellbeing, University of Glasgow, 1 Horselethill Road, Glasgow, G12 9LX, UK

**Keywords:** Child health, General practice, Preventive health services, Health promotion, General practitioners, Health visitors

## Abstract

**Background:**

GPs contribute to preventive child health care in various ways, including provision of child health surveillance (CHS) reviews, opportunistic preventive care, and more intensive support to vulnerable children. The number of CHS reviews offered in Scotland was reduced from 2005. This study aimed to quantify GPs’ provision of different types of preventive care to pre-school children before and after the changes to the CHS system.

**Methods:**

GP consultation rates with children aged 0–4 years were examined for the 2½ years before and after the changes to the CHS system using routinely available data from 30 practices in Scotland. Consultations for CHS reviews; other aspects of preventive care; and all reasons were considered.

**Results:**

Prior to the changes to the CHS system, GPs often contributed to CHS reviews at 6–8 weeks and 8–9 and 39–42 months. Following the changes, GP provision of the 6–8 week review continued but other reviews essentially ceased. Few additional consultations with pre-school children are recorded as involving other aspects of preventive care, and the changes to CHS have had no impact on this. In the 2½ years before and after the changes, consultations recorded as involving any form of preventive care accounted for 11% and 7.5% respectively of all consultations with children aged 0–4 years, with the decline due to reductions in CHS reviews.

**Conclusions:**

Effective preventive care through the early years can help children secure good health and developmental outcomes. GPs are well placed to contribute to the provision of such care. Consultations focused on preventive care form a small minority of GPs’ contacts with pre-school children, however, particularly since the reduction in the number of CHS reviews.

## Background

Children’s earliest experiences profoundly influence their long term health and development [1-3] and access to effective preventive child health care has been acknowledged as important for many years [4-6]. The UK National Health Service (NHS) offers a core service of proactive care through the universal child health programme (CHP). This comprises certain screening procedures; routine childhood vaccinations; surveillance of children’s growth and development; and provision of age appropriate health promotion and parenting advice [7]. The surveillance and advice components take place in child health surveillance (CHS) reviews offered to all children at specified ages. Practice nurses (PNs) and health visitors (HVs) usually have lead responsibility for delivery of vaccinations and child health surveillance reviews respectively but general practitioners (GPs) also provide substantial input to delivery of the universal elements of the CHP. In some practices, GPs retain responsibility for provision of vaccinations, and in almost all practices GPs provide at least some elements of the CHS reviews.

Beyond these core universal services, there is a complex web of additional preventive care provided to young children with particular needs due to health, developmental, or wider social issues. Health visitors often lead delivery of this additional preventive care but again GPs also make an important contribution [8]. GPs provide detailed assessment of children suspected of having a medical, developmental, or family wellbeing problem; directly provide medical care for these children; and refer on to specialist care as required. As GPs frequently see families with young children, they also play an important role in provision of opportunistic preventive care and alerting relevant colleagues to families that could benefit from additional support [9].

The Royal College of Paediatrics and Child Health periodically issues recommendations on the content and delivery of the CHP. The latest report, Health for all Children 4 (HFAC4), was published in 2003 [10]. One of its key messages was that the core programme of CHS reviews should be reduced to allow more flexible and intensive preventive care and support to be offered to families with additional needs. HFAC4 has influenced child health policy across the UK [11-13]. The linked policy in Scotland, published in 2005, went further than HFAC4 in recommending a marked reduction in the number of universal CHS reviews provided to pre-school children, from six (at 10 days; 6–8 weeks; and 8–9; 22–24; 39–42; and 48–54 months) to two (at 10 days and 6–8 weeks), with a selective review at 24 months for children thought to need it due to additional needs or vulnerability [13]. In Scotland the 10 day review is always provided as a home visit by the Health Visitor but GPs can potentially be involved in the provision of any of the other reviews, either on a routine basis or on an ‘as required’ basis if requested by the HV.

The 2005 policy gave NHS Boards across Scotland some flexibility regarding when to implement the revised programme of CHS reviews and the implementation date in different Boards consequently varied between 2005 and 2010. NHS Boards offered the traditional schedule of CHS reviews right up to the date of implementation in their area, then the revised schedule from the implementation date onwards [14]. The changes to the CHS system in Scotland were implemented without piloting or any central plans for evaluation.

This study aimed to explore the following questions using routinely available GP consultation data:

To what extent were GPs in Scotland involved in the delivery of CHS reviews for pre-school children before and after the changes to the CHS system?

To what extent were GPs involved in the delivery of other preventive care to this age group before and after the changes to the CHS system?

What proportion of GP consultations with pre-school children is focused on preventive care and how has this changed over time?

## Methods

GP consultation data were obtained from the NHS Scotland Information Services Division (ISD) Practice Team Information (PTI) system [15]. Under the PTI system, a sample of GP practices from across Scotland, that together are broadly representative of all practices, return data on all face to face GP consultations. Participation in the PTI system is voluntary, and practices are free to join and leave at any time. At any one time, around 60 practices serving around 5% of the Scottish population contribute to the scheme. Data captured on each consultation include patient demographics and Read codes for one or more aspects (symptom, sign, diagnosis, or scheduled care event) of the consultation.

For this analysis, the 30 practices that submitted complete GP consultation data from 1 April 2003 to 31 March 2010 and were in an NHS Board area that implemented the revised CHS system on a specified date prior to mid 2007 were included. The included practices had a combined list size of 200,852 on 1 April 2010, including 11,214 children aged 0–4 years. Practices were drawn from 10 of the 14 NHS Board areas across Scotland, were of a range of sizes (list size from around 4,000 to around 19,000), and served a range of affluent/deprived and urban/rural areas. The revised CHS system was implemented in the included practices’ NHS Board areas on dates ranging from 1 Oct 2005 to 1 May 2007. Consultations for each practice occurring during the 2½ years (10 sequential quarters) before and after the implementation date were included in the analysis.

Consultations for the reasons shown in Table 1 were identified using specially developed lists of Read codes. The lists were specified after:

Review of relevant (previously developed) ISD Read code groupings (e.g. ‘child health care’).

Supplementary manual searching of Read code version 2 (Scottish) browser.

Survey of practices. To confirm that all relevant codes relating to provision of CHS reviews had been captured, a survey of practices was undertaken. The largest practice from each NHS Board area was sent an email survey in February 2011: 8 out of 10 responded. The survey asked about GP contribution to specific child health reviews before and after implementation of revised CHS and which Read codes were assigned to the relevant consultations.

Review by relevant colleagues. The final code lists were reviewed for completeness and accuracy by a Consultant in Public Health Medicine with expertise in health information and maternal and child health and a specialist clinical coder.

**Table 1:** Consultations included in the analysis

**Broad category**	**Subcategory**
Child health reviews	6-8 weeks
	8-9 months
	21-24 months
	39-42 months
	48 months/pre-school
	Scheduled reviews of pre-school children at other specified ages
Other preventive care consultations	Postnatal care (including examination of newborn)
	Immunisation (all universally offered pre-school vaccinations)
	Medical and developmental assessment (eg examination of hips or heart or any aspect of development)
	Health promotion advice and parenting support (eg provision of advice on child safety or behaviour or parental support)
	Assessment and advice relating to child nutrition and growth (eg advice on breastfeeding or weaning or child growth monitoring)
	Child protection (eg child ‘at risk’ or neglected/abused)
Other consultations	Any other reason
Total	All consultations

Notes:

For most consultation types, only consultations with children aged 0-4 years were examined. For the following consultation types, consultations with women aged 15-49 years were also (separately) examined. Restricted code lists were used to ensure only consultations relating to children were included.

Postnatal care.

General health advice and parenting support.

Assessment and advice relating to child nutrition and growth.

Code lists finalised June 2011 using Read code version 2 (Scottish) browser. Full details of the Read codes used are provided in the Appendix.

The codes indicating child health reviews were divided into subcategories indicating each of the specific reviews offered prior to the change in CHS that GPs were potentially involved in and an additional subcategory of ‘scheduled reviews of pre-school children at other specified ages’. This last subcategory included all other codes indicating reviews at different ages at which universal reviews were not usually offered. All ‘other preventive care’ consultations, and those that were not also coded as a child health review (i.e. those that represented additional consultations), were identified separately. For relevant subcategories of ‘other preventive care’ (postnatal care; health promotion advice and parenting support; and assessment and advice relating to child nutrition and growth), consultations with women aged 15–49 were also examined since maternal consultations may be for preventive care of young children. Restricted code lists were used to identify consultations with women to ensure that only relevant consultations were picked up: all codes lists are provided as an Appendix.

Practice population figures at the end of September for every year studied were used to give approximate list sizes for the preceding April to the subsequent March. Consultation rates per 1,000 children aged 0–4 years (or women aged 15–49 years where appropriate) were then calculated for each practice individually and all practices combined for 10 sequential quarters pre- and post-implementation of the changes to the CHS system.

Analysis for this study was conducted within the NHS Scotland Information Services Division and no patient identifiable data were involved. PTI practices are informed by ISD that the data they submit will be used in anonymised form for routine NHS publications and research purposes, and practices are made aware of research outputs based on PTI data. No ethical approval was required for this study (confirmed by the West of Scotland Research Ethics Committee). ISD’s Caldicott Guardian confirmed that the analysis for this study was within normal ISD practice and no additional permissions were required.

## Results

### Scheduled child health reviews

**Figure 1:**
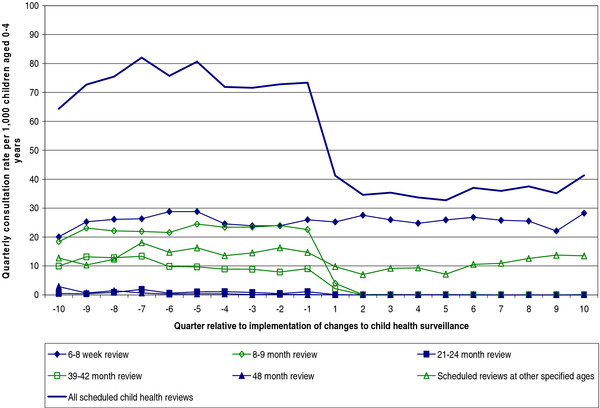
GP consultations with children aged 0–4 years for child health reviews, rates before and after implementation of changes to the child health surveillance system.

Prior to the changes to CHS, the commonest child health review recorded as being provided (at least in part) by GPs was the 6–8 week review (average quarterly consultation rate of 25.4 per 1,000 children aged 0–4 years for the 10 quarters prior to the change in CHS). GP provision of the 8–9 month review was slightly less common (22.5 consultations per 1,000 children 0–4 years per quarter) with provision of the 39–42 month review (10.3 consultations per 1,000 children 0–4 years per quarter) and reviews at ‘other specified ages’ (14.4 consultations per 1,000 children 0–4 years per quarter) less common still. Very few GP consultations were coded as 21–24 month or 48 month reviews (Figure 1).

GP provision of 6–8 week reviews was broadly consistent over the period of study (average quarterly consultation rate of 25.8 per 1,000 children 0–4 years for the 10 quarters after the change in CHS). By contrast, there was a sudden, almost complete fall in the provision of all other child health reviews provided at specified ages (8–9, 21–24, 39–42, and 48 months) immediately after the implementation of the revised CHS system (average consultation rates all <0.5 per 1,000 children 0–4 years per quarter). There were essentially no GP consultations coded as 21–24 month reviews after the changes to CHS despite the availability of the selective 24 month review during this period which would have been identified by the codes used. Consultations for child health reviews at other, non-standard, ages dropped slightly around the time the CHS schedule was changed before increasing back to previous levels (average consultation rate of 10.4 per 1,000 children 0–4 years per quarter).

### Other preventive care

**Figure 2:**
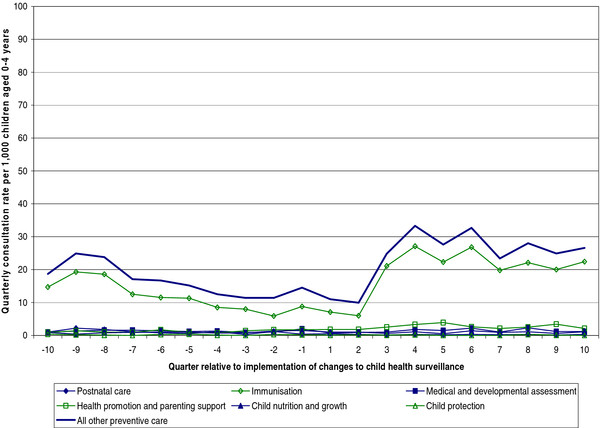
Additional (non-child health review) GP consultations with children aged 0–4 years for other preventive care reasons, rates before and after implementation of changes to the child health surveillance system.

Across the study period there were consistently few additional (i.e. non-child health review) GP consultations with children aged 0–4 years recorded as being for the various types of ‘other preventive care’, with the exception of the immunisation subcategory (Figure 2). Overall, consultations for immunisation steadily declined over the first part of the study period then sharply increased around six months after the changes to CHS. More detailed examination of the rates for the individual practices show that this overall trend was driven by two practices with sharply declining rates early in the period of study and two other practices with sharply increasing rates over the latter part of the study. Additional consultations for child protection were consistently particularly uncommon.

The majority of consultations coded to the various subcategories of ‘other preventive care’ (overall 87%) were not also coded as a child health review hence trends were very similar whether all ‘other preventive care’ consultations, or only those that were additional to child health reviews, were examined. Consultation rates with women aged 15–49 years for postnatal care; health promotion advice and parenting support; and assessment and advice relating to child nutrition and growth also showed no change around the time the revised CHS system was implemented.

### All consultations

**Figure 3:**
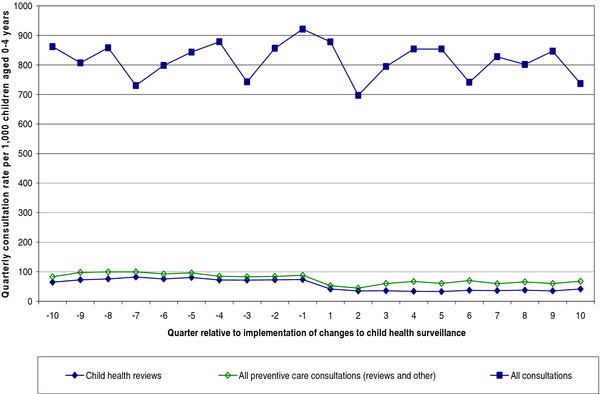
Relative contribution of different types of GP consultations with children aged 0–4 years, rates before and after implementation of changes to the child health surveillance system.

The overall GP consultation rate with pre-school children was relatively constant over the period of study, with some seasonal periodicity evident (Figure 3). Child health reviews and, in particular, additional consultations coded as other types of preventive care, form a small proportion of all GP consultations with children aged 0–4 years. In the 2½ years before and after the changes to CHS, all preventive consultations combined accounted for around 11% (9,606 / 87,938) and 7.5% (6,709 / 88,698) respectively of all consultations with this age group, with the decline due to reductions in GP provision of child health reviews.

## Discussion

We have used routinely available consultation data to explore GPs’ contribution to the preventive care of pre-school children, and to examine the impact of the changes to the child health surveillance system that were implemented in Scotland from 2005.

Prior to the changes to the CHS system, GPs made a substantial contribution to the provision of child health reviews, particularly those offered at 6–8 weeks and 8–9 months (and to a lesser extent 39–42 months) of age. Following the changes, GPs have continued their involvement in the 6–8 week review but provision of other standard reviews has essentially ceased. This finding is broadly in line with what would have been expected from the policy recommendations, although it is worth noting that policy is by no means always implemented as intended [16,17]. Our findings also show that, since 2005, GPs have had minimal involvement in provision of the selective 24 month review. This is perhaps not surprising as GPs historically had little involvement with the universally provided 21–24 month review, but it does suggest that GPs now have minimal input into proactively assessing children’s development after early infancy.

Despite extensive code lists, relatively few additional (non-child health review) GP consultations with pre-school children for other aspects of preventive care were identified. Changes over time in the number of GP consultations involving childhood immunisations appear to reflect occasional changes in GP provision of routine immunisations in individual practices rather than any specific effect of the changes to the CHS system on GP involvement in this aspect of children’s care. Consultations coded as involving child protection were noticeably rare, particularly in light of evidence that unhelpful parenting, neglect, and abuse are very common and have serious implications for children’s outcomes [18]. In general, changes to the CHS system appear to have had minimal, if any, impact on GPs’ provision of these wider aspects of preventive child health care. In particular, there is no evidence that withdrawal of ‘routine’ child health reviews has led to an increase in the number of non-child health review consultations for pre-school children that are focused on preventive care. Our results cannot comment on whether or how the characteristics of pre-school children receiving preventive consultations have changed over time however.

This study involved 30 practices from across Scotland that together serve over 11,000 pre-school children. The PTI information system is well established and subject to ongoing data quality assurance procedures [15]. PTI practices are asked to code all the clinical findings/activity relevant to each consultation as precisely as possible using as many Read codes as necessary and GPs usually assign the Read codes themselves during the course of their consultations. The Read code lists used in this analysis were carefully specified to reflect the range of preventive child health care that GPs may be involved in and all recorded codes were included in the analysis. The codes assigned to a consultation will be those considered necessary by a GP for safe and effective care hence may not reflect all aspects of the consultation. It is likely that some opportunistic health promotion activity will not have been recorded and therefore not reflected in this analysis. Consultations that had provision of preventive care as a substantive component should have been identified however and the trends (or lack of them) identified are likely to be genuine.

This study has specifically examined changes over time in the preventive care delivered to pre-school children by GPs. Preventive health care provided by GPs is only one element of the complex system of services that aims to protect and promote young children’s health and development however, with Health Visitor and early education/childcare services amongst others also being important. A separate national information system, Child Health Surveillance Programme – Pre School (CHSP-PS) collects information on completed child health reviews from Health Visitors but this system does not record information on all contacts between HVs and young children [14]. The PTI system did collect information on all Health Visitor consultations with the practices’ patients from 2003/04 but this data collection stopped in 2005/06 hence PTI data cannot provide information on how the totality of HV consultations with pre-school children changed after the changes to the CHS system [15].

It is known from the CHSP-PS data that HVs also ceased universal provision of child health reviews after 6–8 weeks after implementation of the 2005 policy [14,19], hence our results reflect a genuine withdrawal of these later universal reviews rather than just a shift in their delivery from GPs to HVs. Since implementation of the revised CHS system, HVs have provided the selective 24 month review to around 25% of children, although GPs have had minimal involvement in this review as noted above.

The changes to the Scottish CHS system were explicitly designed to free up existing HV time to focus available resources on children most in need of preventive care. The lack of data on care apart from routine child health reviews provided by Health Visitors means that the overall impact of the changes to the CHS system on the amount, content, and distribution of HV care (and how this relates to changes in GP provision of preventive care) therefore cannot be directly assessed. Some local areas are starting to use electronic HV case record systems which may in time make more detailed analysis of HV activity, and hence a more complete assessment of the preventive care provided to young children, feasible.

The configuration of the child health surveillance system has been the subject of longstanding debate [5]. The question of how many universal reviews are required, and at which ages, to form an effective and efficient service through which to reliably deliver early identification of health and developmental problems and provide universally relevant health promotion advice and parenting support, and from which to target additional support to families most in need, continues to exercise policy makers. Some elements of the child health programme (for example neonatal hearing screening, immunisation, and certain aspects of the CHS reviews such as provision of advice on reducing the risk of sudden infant death syndrome) are supported by high quality evidence, but in general robust evidence that directly answers detailed service organisation questions is lacking. The HFAC reports are therefore explicitly based on drawing together multiple stands of different types of evidence along with consensus professional opinion to provide the best possible recommendations given the evidence available. It is notable that the revised CHS system implemented in Scotland from 2005 onwards has delivered a considerably reduced schedule of pre-school child health reviews compared to that recommended in HFAC4.

This study did not set out to investigate the impact of the changes to the CHS system on young children’s outcomes although ultimately securing equitable positive health and developmental outcomes for children is the goal of the preventive care system. There is some evidence that the changes to the Scottish CHS system implemented from 2005 have compromised the early detection of some developmental problems. An audit in one NHS Board area suggested that the age of children referred to speech and language therapy increased considerably after the changes, and a separate pilot project looking at reinstating universal developmental reviews for toddlers found a large number of children with previously undetected developmental delays [20]. This evidence is clearly limited (and it is not possible to comment on whether changes in GP provided care have made a specific contribution to the changes seen) but comprehensive data on the detection of childhood developmental problems are lacking, making more definitive assessments difficult. Nevertheless, in response to concerns about the impact of the CHS changes on the overall functioning of the preventive care system, the Scottish Government has recently recommended the introduction of a new 24–30 month child health review for all children, although this is yet to be fully implemented [21,22].

## Conclusions

GP provision of universal child health reviews has fallen considerably in Scotland since implementation of the revised child health surveillance system from 2005 as would have been expected. Since 2005, GPs have also had minimal involvement in the selective child health reviews provided by Health Visitors to vulnerable toddlers: this raises questions about the adequacy of developmental and physical health assessments being provided to this age group.

Additional (non-child health review) GP consultations with young children for any aspect of preventive care (except immunisation in some practices) are uncommon, with consultations recorded as involving child protection virtually non-existent, and the changes to child health surveillance system have had no obvious impact on provision of these additional consultations. GPs are well placed to make an important contribution to the overall preventive care of young children by promoting positive family relationships; supporting parenting; providing consistent, evidence based guidance on issues such as child nutrition; and recognising and intervening swiftly when children’s health or development is at risk [23]. The relatively low proportion of GP consultations with young children that is focused on preventive care suggests it may be debatable whether this potential is being fully realised at present.

## Abbreviations

CHP, Child health programme; CHS, Child health surveillance; CHSP-PS, Child health surveillance programme – pre-school (Scottish national information system that provides information on delivery of child health reviews); GP, General Practitioner; HFAC4, Health for all children 4; HV, Health Visitor (community nurse with particular responsibility for preventive child health); ISD, NHS Scotland Information Services Division; NHS, National Health Service; PN, Practice Nurse (GP employed nurses providing a range of ‘treatment room’ services to patients of all age groups); PTI, Practice Team Information (Scottish national information system that provides information on GP and PN consultations); UK, United Kingdom.

## Competing interests

No author has a financial or non-financial conflict of interest to declare.

## Authors’ contributions

RW led on study design and data analysis and wrote the first draft of the manuscript. PW provided substantive input on study design, Read code selection, interpretation of findings, and revision of manuscript drafts. Both authors read and approved the final manuscript.

## Authors’ information

Rachael Wood – corresponding author, Honorary Clinical Senior Lecturer, Centre for Population Health Sciences, University of Edinburgh, Teviot Place, Edinburgh EH8 9AG, rachael.wood@ed.ac.uk and Consultant in Public Health Medicine, Information Services Division, NHS National Services Scotland, Gyle Square 1 South Gyle Crescent, Edinburgh EH12 9 EB

Philip Wilson Senior Lecturer, Institute of Health and Wellbeing, General Practice and Primary Care, University of Glasgow, 1 Horselethill Road, Glasgow G12 9LX, philip.wilson@glasgow.ac.uk

## Funding

Rachael Wood undertook this work whilst in receipt of a Clinical Academic Training Fellowship from the Chief Scientist Office for Scotland (CAF/06/05).

## Pre-publication history

The pre-publication history for this paper can be accessed here:

http://www.biomedcentral.com/1471-2296/13/73/prepub
